# All-cause mortality according to COVID-19 vaccination status: An analysis of the UK office for National statistics public data

**DOI:** 10.12688/f1000research.154058.2

**Published:** 2025-02-20

**Authors:** Marco Alessandria, Giovanni Malatesta, Giovanni Di Palmo, Marco Cosentino, Alberto Donzelli

**Affiliations:** 1 Department of Life Sciences and Systems Biology, University of Turin, Torino, Italy; 2 Physics Graduate, Member of the Scientific Committee of the Fondazione Allineare Sanità e Salute, Pistoia, Italy; 3 B.E., Data Analysis Specialist, Taranto, Italy; 4 Center for Research in Medical Pharmacology, University of Insubria, Varese, Italy; 5 MD, Specialist in Hygiene and Preventive Medicine, independent Medical-Scientific Commission; President, Fondazione Allineare Sanità e Salute, Milano, Italy

**Keywords:** COVID-19; COVID-19 vaccinations; all-cause mortality; Standardized Mortality Ratio

## Abstract

**Background:**

The mass vaccination campaign against COVID-19 has been commonly considered the best response to the global COVID-19 pandemic crisis. However, assessment of its real-world effect can be performed by analysis of all-cause mortality by vaccination status. The UK is perhaps the only country which has made publicly available all-cause mortality data by vaccination status.

**Methods:**

Data from April 2021 to May 2023 published by the UK Office for National Statistics (ONS) were retrospectively analyzed by age groups and vaccination status; the standardized mortality ratio (SMR) for all-cause and non-COVID-19 mortality was calculated against the corresponding unvaccinated groups.

**Results:**

We found that across all age groups, all-cause mortality SMRs increased from a certain date, dependent on the age group. Across all age groups, all-cause mortality SMRs were initially much lower than 1. However, due to their increase, by a certain date for the 18-39, 80-89 and 90+ age groups they exceeded the reference value. For the other age groups, the date at which the SMR would reach 1 can be predicted, provided the trend is maintained. Non-COVID-19 SMRs’ trends were very similar. Their initial values much lower than 1 are suggestive of significant biases in the ONS dataset, leading to underestimate the risks for the vaccinated people, as it is implausible that COVID-19 vaccines protect against non-COVID-19 deaths.

**Conclusions:**

The increase over time in all-cause death SMRs in vaccinated people compared to unvaccinated, and their excess from the reference values for certain age groups, should be carefully considered to understand the underlying factors. Furthermore, since the initial values of the SMRs are much lower than 1, we assume the presence of significant biases in the ONS dataset, leading to understimate the risks for the vaccinated people, as it is implausible that COVID-19 vaccines protect against non-COVID-19 deaths. It would be desirable for other major countries to systematically collect all-cause mortality by vaccination status and, in the meantime, a pending indepth investigations, much greater caution should be exercised in promoting mass vaccination campaigns.

## 1. Introduction

Due to the COVID-19 pandemic crisis and the subsequent COVID-19 mass vaccination campaign, the interest has hugely soared in publicly available data on all-cause mortality. For example, data from England and Wales show in 2022, in comparison to the previous average five-year reference period, an excess mortality with a trend driven by more deaths than expected starting in March 2022. ^1^ From April 2021 to the end of May 2023 (the period covered by the ONS dataset) the total excess mortality amounted to 129,801 deaths above the five-year average. ^2^ A similar trend occurs in many other countries in the European Union (EU), as indicated by the graphs and maps provided by the European Mortality Monitoring Project (EuroMoMo), a routine public health mortality monitoring system aimed at detecting and measuring excess deaths related to public health threats across EU countries. According to EuroMoMo, the excess deaths in 2022 were 328,047 in 2022 and 305,301 in 2021 ( Graphs and maps — EUROMOMO, accessed May 1, 2024). This is clearly an anomaly, as previous mortality shocks over the past 120 years have almost always been followed by immediate rebounds back, in one to two years, ^3^ with normalization of mortality risk.

A similar observation has also been made ^4^ about excess mortality across 47 countries in the Western World since the COVID-19 Pandemic, based on ‘Our World in Data’ estimates of January 2020 to December 2022. Indeed, excess mortality was registered in 87% of countries in 2020, in 89% in 2021 and in 91% in 2022. During 2021, when not only containment measures but also COVID-19 vaccines were used to tackle virus spread and infection, the highest number of excess deaths was recorded. ^4^

England and Wales benefit from one of the best public health data collection systems in the world, and are therefore uniquely positioned to monitor and investigate the above-mentioned phenomenon. ^1^ Moreover, the Office for National Statistics (ONS) of the United Kingdom (UK) has published all-cause mortality data in England, ^5^ stratified according to COVID-19 vaccination status, thus overcoming the intrinsic limitation of just identifying deaths due to COVID-19, as for instance happens so far in Italy and in most if not all EU countries, and allowing a direct assessment of the eventual consequences of COVID-19 vaccination for individual as well as public health in terms of change not only of COVID-19 mortality but also of all-cause mortality. In addition, the UK vaccinated more than 50% of its eligible population in the first four months of 2021 and by the end of 2021, 77% received at least one dose, ^6^ thus exceeding the aforementioned threshold earlier than most of the other EU countries. It is therefore possible that the trends observed in England and Wales anticipate what will later occur in EU.

We decided therefore to analyze the ONS public data on all-cause mortality according to vaccination status, starting from the rates already officially provided by the ONS itself on its website. ^5^ We calculated the rate ratios RR by vaccination status for every age group. Furthermore, due to month-to-month variation in the populations of individual vaccination status, we decided to calculate Standardized Mortality Ratios (SMRs) for those vaccinated with any dose in the different age groups, and to evaluate any potential emerging trends over time. A previous version of this study, dealing with UK ONS data from January to May 2021, is available on Preprints.org. ^7^

## 2. Methods

In this retrospective study we collected data from the UK ONS web-based platform. ^5^ This platform gathers total mortality data by vaccination status from April, 2021 until May, 2023. Data are publicly available under the Open Government license ( https://www.nationalarchives.gov.uk/doc/open-government-licence/version/3/, accessed on 12 February 2024), and can therefore be freely analyzed and published provided that the source is properly acknowledged.

Relying on the excel file provided by this platform, we utilized data from the spreadsheet named “Table 2”(extended data), inasmuch as, differently from other spreadsheets, it provides proper stratification by age and vaccination status to perform an estimate of the Standardization Mortality Rate (SMR) and Relative Risks (RR) for the All-causes death and Non-COVID-19 deaths variable. We could not consider Deaths involving COVID-19 inasmuch from the UK ONS dataset, the absolute frequency of deaths in many vaccination statuses and many age group indicated for this variable was <3 mostly for the younger age groups and for many months of the year 2023. We were therefore unable to reliably calculate the RRs and SMRs. The spreadsheet used for this analysis provides seven age groups (18–39, 40–49, 50–59, 60–69, 70–79, 80–89, 90+ years) and each age group is further subdivided into several classes based on vaccination status:

• Unvaccinated,
• First dose less than 21 days before (1D<21d),
• First dose at least 21 days before (1D≥21d),
• Second dose less than 21 days before (2D<21d),
• Second dose at least 21 days before (2D≥21d),
• Third dose or booster less than 21 days before (3D<21d),
• Third dose or booster at least 21 days before (3D≥21d).

Even if the ONS marks with a “u” (unreliable) any rates arising from a number of death lower than 20, nonetheless we decided to consider groups with a minimum of 10 deaths. Though being aware that the lower the number of deaths, the greater the uncertainty on both the rates and the RRs, this choice allowed us to identify trends of RR over time for each age group. Given the extremely variable nature of the RR trend over time, we decided to understand if this phenomenon was related to the distribution of populations between the various vaccination statuses and for each month of observation. In this regard, we created stacked graphs, in order to analyze the population distributions of the vaccination status for each age group and for each month of the entire observation period, inserting on the y-axis the person-years and on the x-axis the observation months (Supplementary materials, Tables S1-S7(extended data) and Figures S1A/B-S7A/B)((extended data)). From the stacked graphs, a dynamic distribution emerged over the entire observation period for all vaccination statuses. Furthermore, for all age groups was observed an almost constant distribution of the Unvaccinated population over period, unlike the 18-39 and 40-49 age groups where, for the first months of the observation, these groups were more representative compared to other vaccination status. Based on these observations, in order to manage the dynamic distribution of the vaccination statuses month per month, we decided to calculate the SMRs for each observation month. From the calculation of the SMRs boxes indicating <3 deaths were excluded. Finally, for the 18-39, 40-49 age groups we decided do not consider the first six months and the first three months respectively while for the 50-59 we did not consider the first month, in order to compare a roughly constant distribution over time of the Unvaccinated population with the Vaccinated population and obtain a reliable estimate of the SMRs. This decision was made considering a percentage variation of no more than 1% between months in the unvaccinated population. Subsequently, we investigated the relation between SMRs and observation months applying a simple regression model and using SMRs as dependent variable and the observation months as independent variable. Finally, we calculated the intersection of the regression line with the reference line for the unvaccinated (y=1) to identify where possible, or predict where not, the moment in which deaths from all causes in the vaccinated group exceed those of the unvaccinated.

### 2.1 Statistical analysis

To calculate the relative risk (RR) between the vaccinated and unvaccinated populations, we used the age-standardized rates indicated in the excel files provided by the UK ONS. ^5^ Their 95% confidence intervals (CI) were calculated according to the following formula ^8^^,^^9^:

$${\mathrm{CI}}_{95(\mathrm{RR})}=e^{\left[\ln\left(\mathrm{RR}\right)\pm 1.96*{\mathrm{SE}}_{\ln\left(\mathrm{RR}\right)}\right]},$$

where “ln (RR)” is the natural logarithm of the Relative Risk and “SE _ln(RR)_” is the standard error of the natural logarithm of the RR.

The SE _ln(RR)_ was calculated for each vaccination status of each of the age groups according to the formula:

$$\text{SEln}\left(\mathrm{RR}\right)=\sqrt{\left(\frac{V.\mathrm{Pop}.-\text{Stand}.D.\mathrm{Exp}.}{V.\mathrm{Pop}.*\text{Stand}.D.\mathrm{Exp}.}\right)+\left(\frac{\mathrm{Un}.\mathrm{Pop}.-\text{Stand}.D.\mathrm{Exp}.}{\mathrm{Un}.\mathrm{Pop}.*\text{Stand}.D.\mathrm{Exp}.}\right)},$$ (1)

Where, for each year, “V.Pop.” represents the vaccinated population, “Un.Pop.” represents the unvaccinated population, and “Stand.D.Exp.” represents the expected standardized deaths, that is, the deaths that would occur by applying the Age-standardised mortality rates per 100,000 person-years (Note 1, spreadsheet “Note” UK ONS web-based platform ^6^) to the real population, calculated according to the formula:

$$\text{Expected Standardized Deaths}=\frac{\;\mathrm{Age}-\text{standardized rate}*\text{Population of each vaccine status}}{100.000}.$$

The choice to use the “Expected Standardized Deaths” is justified by the fact that the calculated RR expresses the ratio between two standardized rates based on the European population. The P value was calculated according to Altman and Bland ^10^:

$$p=\exp\left(-0.717\times z-0.416\times z^{2}\right)$$

where $z=\frac{\ln\left(\mathrm{RR}\right)}{\mathrm{SE}}$ and SE is the (1).

To calculate SMR indirect standardization method was applied according to Naing (2000). ^11^ Before to use the simple regression model, all the assumption of the model was verified: scatter plot was created to verify the linear relationship between variables, Shapiro-Wilk normality test was used to verify the residual distributions and Breusch-Pagan test was used to verify homoscedasticity of the variance of errors. To calculate the intersection of the regression line with the reference line of the unvaccinated for each age group we indicated the observation months with a progressive number and solved the equation of the regression line for x and assigning y=1. The x value obtained was compared with the numbers assigned of the observation months so that we could identify a specific moment in the observation period.

Data was processed using R studio (version 2023.09.0).

## 3. Results

### 3.1 About ONS dataset

The UK ONS dataset we investigated is the latest version available. ^5^ It is based on the population in Census 2021, linking Census deidentified records to National Health Service (NHS) numbers. People with no NHS number or multiple entries are not included.

The individuals were then linked via NHS number to vaccination data from the National Immunisation Management Service (NIMS) and ONS death registrations. The population was restricted to people in England, alive on 1 April 2021. Overall, ONS dataset population (51,786,812 people) covers 91.6% of the England population on Census Day 2021. The excluded population therefore amount to almost 4,600,000 people. Furthermore, 103,142 were excluded due to erroneous or inconsistent vaccination data, so the overall excluded population amounts to almost 4,700,000 people.

Finally, of the 1,149,784 deaths that occurred in England between 1 April 2021 and 31 May 2023, 90.6% (1,041,524) could be linked to individuals in the 2021 Census.

### 3.2 Population distributions

The stacked graphs showed a dynamic distribution over all observation period whose percentages and absolute frequencies are reported in Supplementary Material (Tables S1-S7 and Figures S1A/B–S7A/B) (Extended data). ^39^

### 3.3 Rate Ratios (RR)

All-causes mortality rate ratios RR according to vaccination status are shown in Supplementary, Tables S8-S14(Extended data) ^39^ and Figures S8-S14(Extended data). ^39^ Similarly, RRs for non-COVID19 related deaths are shown in Tables S15-S21(Extended data) ^39^ and Figures S15-S21(Extended data). ^39^ Main results for both mortality causes are summarized below.

*Deaths from all causes*:

In all age groups, those vaccinated with the first dose at least 21 days ago have a significantly higher risk of death from all causes than those not vaccinated in almost all months of the entire period, except for the 18-39 age group in which the RRs are significatively higher than 1 in half the months. The average RR values in all age groups are between 1.7 and 2.3, except for the 18-39 years group where the average is 1.5. In the age groups 18-39, 60-69, and older RRs present initial peaks higher than 3, up to a maximum of 5.5 in the 70-79 age group.

As regards those vaccinated with 2 doses at least 21 days ago, in the age groups starting from 60 years, the risk of death from all causes in the initial months is much lower than the unvaccinated, with a tendency to increase. Since around a third of the entire period (between October and December 2021) RRs significantly exceeds the reference value, remaining higher in almost all the remaining months, although not significatively in the last few months for 60-69 and 90+ years age groups.

The RRs for those vaccinated with three doses at least 21 days ago, for the age groups 60-69 years and older, present a growth trend which, starting from values much lower than one, reaches and exceeds the reference value:

• for the 60-69 years age group, the risk of death significantly exceeds that of the unvaccinated in the months of November and December 2022 and remains in the following months not significantly different from that of the unvaccinated;
• for the 70-79 years age group the RR exceeds the reference value in June 2022 and always remains significantly higher, apart from the months of September 2022 and May 2023 where the values are not significative;
• for the 80-89 years age group the RR exceeds the reference value in April 2022, reaches the maximum value (RR = 2.29, CI _95_ = 2.04-2.58) and then stabilizes on values always significantly higher than 1;
• for vaccinated people aged 90 years and over, the reference value is exceeded in April 2022 (RR = 1.13, CI _95_ = 1.02-1.26), then remaining at values always significantly higher than 1, with a maximum of 1.85 in November 2022.

*Deaths non correlated to COVID19*

The rate ratios (RR) from non-Covid causes follow the trends already seen for deaths from all causes, with slightly higher values. Therefore, the above considerations can be repeated.

### 3.4 Standardized mortality ratios

*Age groups*

The results of all regression models performed for the all-causes deaths and non-COVID19 deaths variable and for each age group are summarized in Table 1. Furthermore, in table 1 are indicated the intersection of the regression line with the reference line of the unvaccinated also in the graphs where is not possible to visualize the intersection.

**Table 1.** Coefficient of determination (R2) of the regression analysis of SMRs and observation months and p-value of the regression line for each age group and for the All-causes death and Non-COVID-19 deaths variable; intersection: intersection of the regression line with the reference line for the unvaccinated (y=1).

Age group	Variables	R ^2^	p-value	Intersection
18-39	All-causes death	0.601	<0.0001	January, 2023
	Non-COVID-19 deaths	0.500	0.0005	January, 2023
40-49	All-causes death	0.712	<0.0001	September, 2023
	Non-COVID-19 deaths	0.463	0.0003	April, 2023
50-59	All-causes death	0.734	<0.0001	July, 2024
	Non-COVID-19 deaths	0.609	<0.0001	January, 2025
60-69	All-causes death	0.847	<0.0001	February, 2024
	Non-COVID-19 deaths	0.745	<0.0001	May, 2024
70-79	All-causes death	0.860	<0.0001	January, 2024
	Non-COVID-19 deaths	0.764	<0.0001	May, 2024
80-89	All-causes death	0.784	<0.0001	April, 2023
	Non-COVID-19 deaths	0.706	<0.0001	January, 2023
90+	All-causes death	0.695	<0.0001	September, 2022
	Non-COVID-19 deaths	0.705	<0.0001	May, 2022

**Figure 1.**
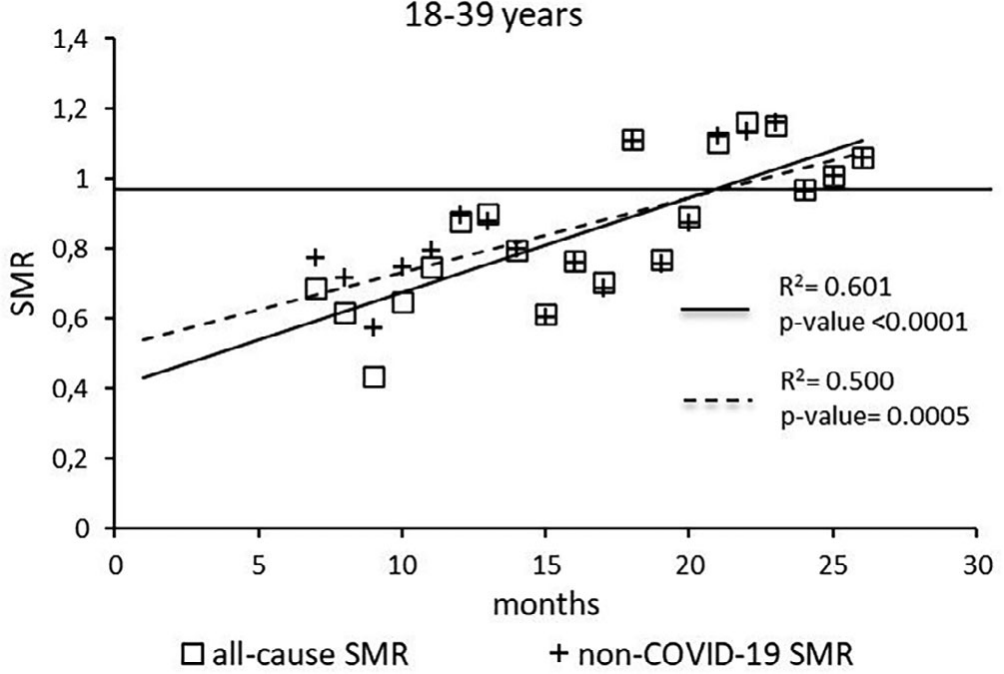
Regression line of the SMR trend of the 18-39 age group.

The regression model for 18-39 age group showed coefficient of determination R ^2^=0.601 and a p-value <0.0001 for the All-causes death variable. For the non-COVID19 deaths regression model showed a R ^2^=0.500 and a p-value=0.0005 ( Figure 1). Both variables showed the intersection of the regression line in January 2023.

**Figure 2.**
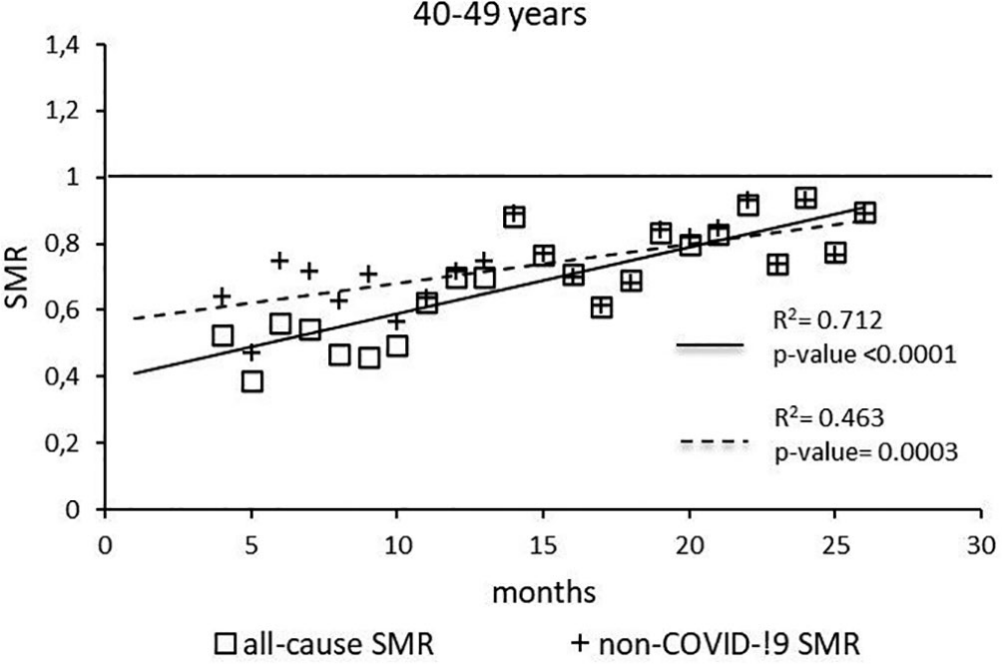
Regression line of the SMR trend of the 40-49 age group.

The regression model for 40-49 age group showed coefficient of determination R ^2^=0.712 and a p-value <0.0001 for the All-causes death variable. For the non-COVID19 deaths regression model showed a R ^2^=0.463 and a p-value=0.0003 ( Figure 2). The All-causes death variables showed the intersection of the regression line in September 2023 while the non-COVID19 deaths showed the intersection of the regression line in April 2023.

**Figure 3.**
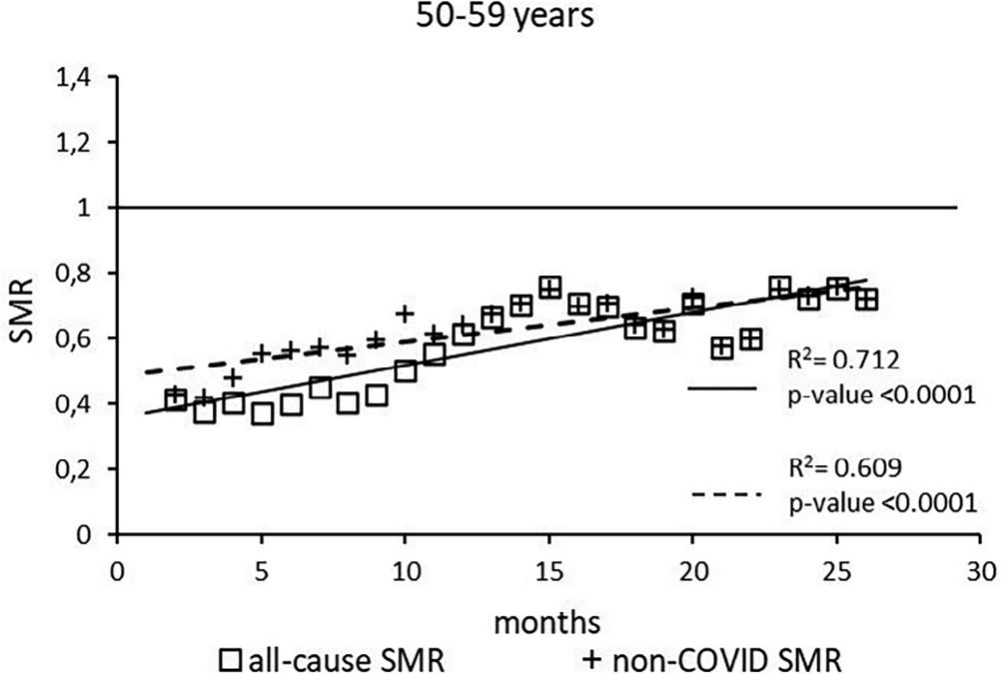
Regression line of the SMR trend of the 50-59 age group.

The regression model for 50-59 age group showed coefficient of determination R ^2^=0.734 and a p-value <0.0001 for the All-causes death variable. For the non-COVID19 deaths regression model showed a R ^2^=0.609 and a p-value<0.0001 ( Figure 3). The All-causes death variables showed the intersection of the regression line in July 2023 while the non-COVID19 deaths showed the intersection of the regression line in January 2025.

**Figure 4.**
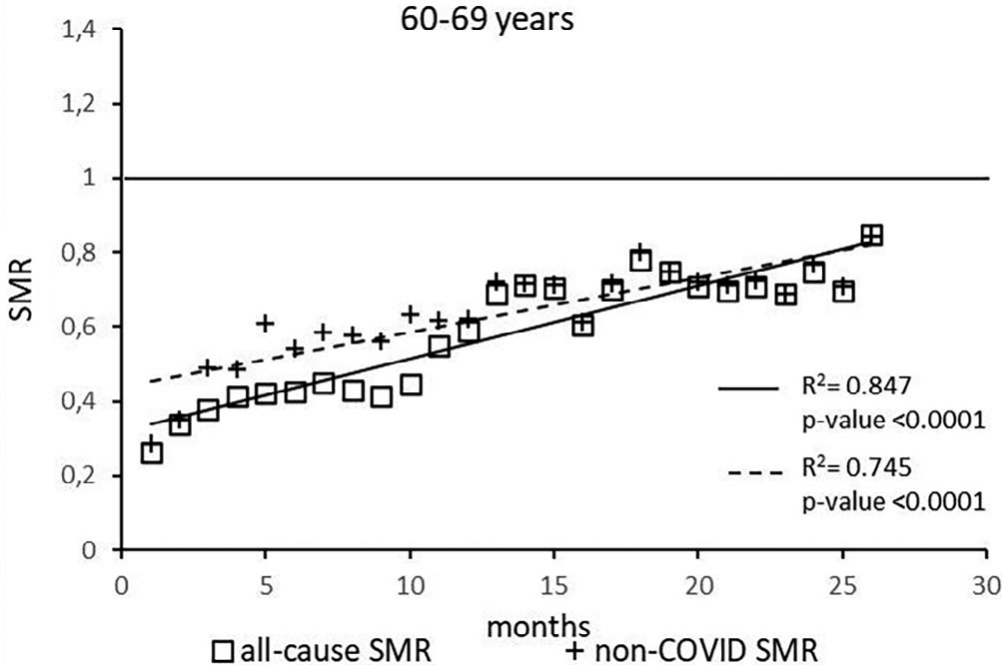
Regression line of the SMR trend of the 60-69 age group.

The regression model for 60-69 age group showed coefficient of determination R ^2^=0.847 and a p-value <0.0001 for the All-causes death variable. For the non-COVID19 deaths regression model showed a R ^2^=0.745 and a p-value<0.0001 ( Figure 4). The All-causes death variables showed the intersection of the regression line in February 2024 while the non-COVID19 deaths showed the intersection of the regression line in May 2024.

**Figure 5.**
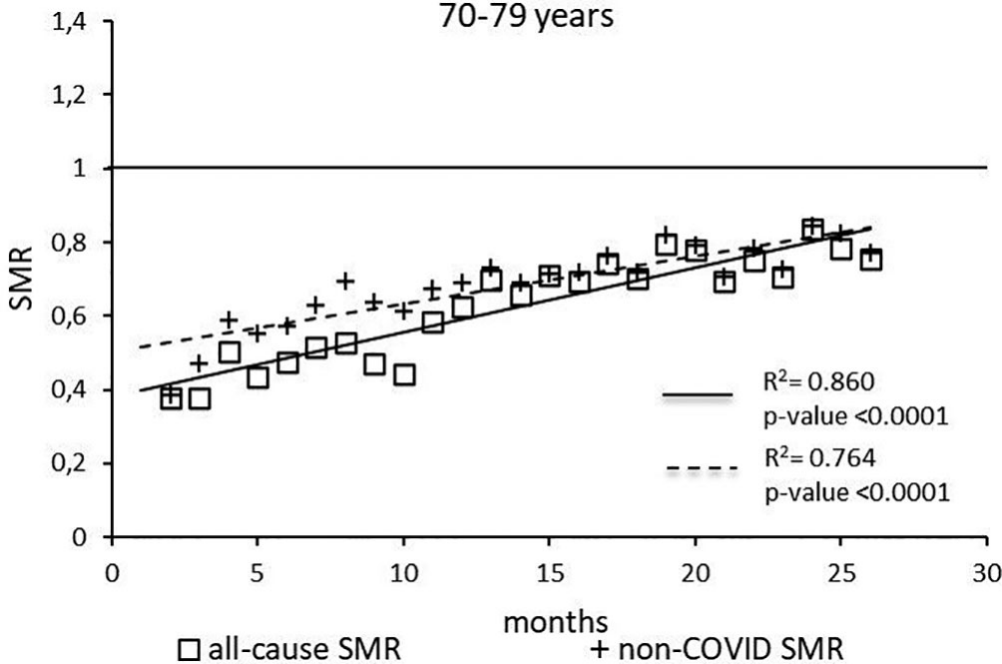
Regression line of the SMR trend of the 70-79 age group.

The regression model for 70-79 age group showed coefficient of determination R ^2^=0.860 and a p-value <0.0001 for the All-causes death variable. For the non-COVID19 deaths regression model showed a R ^2^=0.764 and a p-value<0.0001 ( Figure 5). The All-causes death variables showed the intersection of the regression line in January 2024 while the non-COVID19 deaths showed the intersection of the regression line in May 2024.

**Figure 6.**
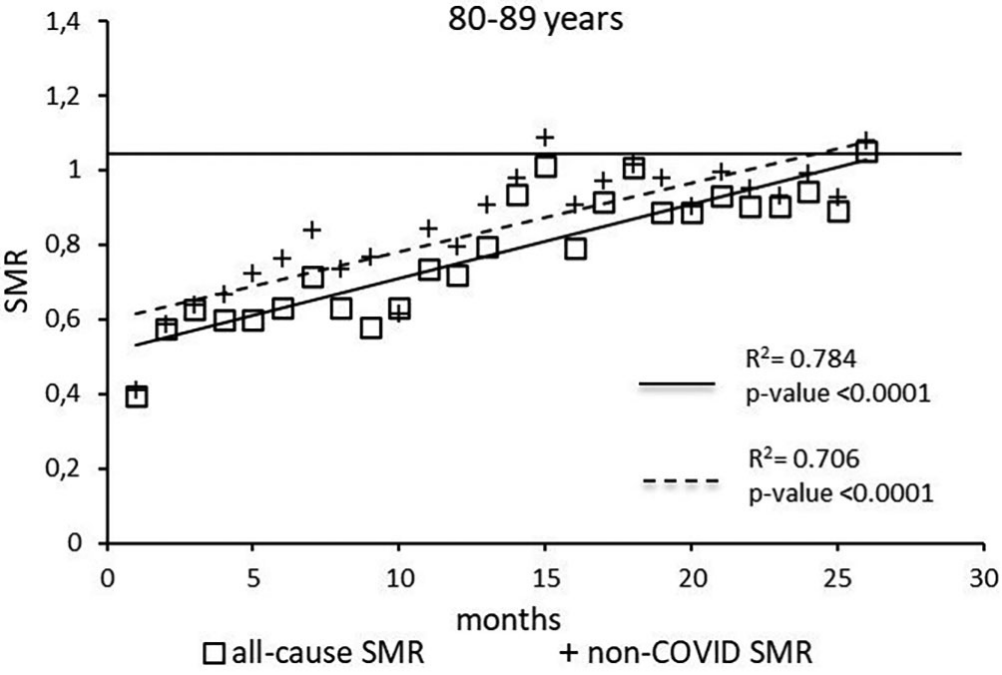
Regression line of the SMR trend of the 80-89 age group.

The regression model for 80-89 age group showed coefficient of determination R ^2^=0.784 and a p-value <0.0001 for the All-causes death variable. For the non-COVID19 deaths regression model showed a R ^2^=0.706 and a p-value<0.0001 ( Figure 6). The All-causes death variables showed the intersection of the regression line in April 2023 while the non-COVID19 deaths showed the intersection of the regression line in January 2023.

**Figure 7.**
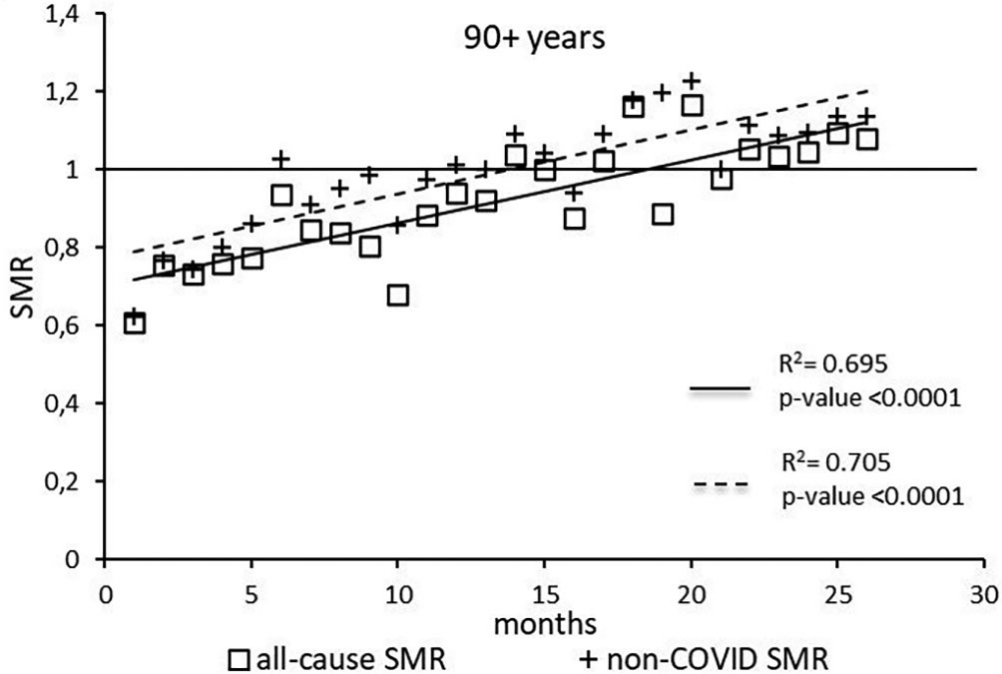
Regression line of the SMR trend of the 90+ age group.

The regression model for 90+ age group showed coefficient of determination R ^2^=0.695 and a p-value <0.0001 for the All-causes death variable. For the non-COVID19 deaths regression model showed a R ^2^=0.705 and a p-value<0.0001 ( Figure 7). The All-causes death variables showed the intersection of the regression line in September 2022 while the non-COVID19 deaths showed the intersection of the regression line in May 2022.

## 4. Discussion

In the present study, we analyzed the UK ONS data on all-cause mortality according to vaccination status, which are publicly available on the ONS institutional website. ^5^ The main findings of our analysis are:

(a) compared with unvaccinated, vaccinated with one or two doses show, in the period April 2021-May 2023, a substantially higher risk of all-causes and non-COVID-19 deaths **.** Indeed, for both causes people vaccinated with one dose show a RR significatively higher than 1 through almost the whole period and in any of the age groups, except the 18-39 years, in which it is significatively higher in half the period. In people who received the second dose, from 60 to 90+ years of age the risk of all-causes and non-COVID-19 death is significatively higher than in unvaccinated people through about the final two thirds of the period. It should be noted that from 50 to 90+ years of age the RRs of all-causes and non-COVID-19 death have implausibly low initial values.
(b) Also vaccinated with three doses have incredibly low initial values of RR both for all-causes and for non-COVID-19 deaths. Their RRs, for people aged 60-69 and older progressively grow up to reach and exceed the reference value from 60 years of age, and from 70 years and over the RRs remain significantly greater than 1.
(c) A linear growth trend is revealed by regression analysis of Standardized Mortality Ratios of people vaccinated with any dose compared to the unvaccinated across all age groups, for both all-cause and non-COVID-19 deaths. The regression lines, both for all-cause deaths and for non-COVID-19 ones, start from very low values for all the age groups. For the age groups 18-39, 80-89 and 90+ years both regression lines intersect the reference line of unvaccinated during the study period. The same occurs for the age 50-59, limited to non-COVID-19 deaths, For the other age groups, we have predicted the month of the intersection (see Table 1).

The results found for the RRs of the first and second doses are confirmed in other studies, e.g. in two studies carried out in an Italian province. ^12^^,^^13^ Both studies show a significantly higher risk of death from all causes for those vaccinated with one and two doses compared to the unvaccinated. Furthermore, they show an implausible high protection of the vaccine against deaths from all causes, too high to be attributed to protection from deaths from COVID-19, which are a minority percentage of total deaths. Both studies, however, are affected by important biases. The main one is the so-called Immortal Time Bias (ITB), as highlighted in an intervention published in an Italian epidemiology journal. ^14^ After correction of ITB, the unlikely protection provided by the third dose against death from all causes disappears entirely. Another recent study, ^15^ was based on the same dataset kindly provided by the authors of the article, ^13^ but corrected for the ITB. In its multivariable analysis, this study shows higher all-cause death hazard ratios (HRs) for individuals vaccinated with one and two doses compared to unvaccinated, and no protection against all-cause deaths for population vaccinated with 3 or more doses. Furthermore, the study ^15^ found a small but statistically significant reduction in life expectancy for vaccinated people with two and three or more doses

The present study also shows extremely low initial risks of all-causes and nonCOVID-19 death, as well in the analysis of the RRs for those vaccinated with two and three doses, as in that of the SMRs for those vaccinated with any dose compared to the unvaccinated. These results appear difficult to justify, especially when referring to non-COVID-19 deaths, unless admitting the presence of some important selection bias. In fact, if the populations being compared were homogeneous, the risk difference of death from causes other than COVID-19 should be about zero, and both the RRs and SMRs for non-COVID deaths should not differ significantly from 1.

Unfortunately, there is a lack of information on the health status of populations or about other factors that influence the risk of death, so we can only formulate some hypotheses, that are not necessarily alternative.

### 4.1 Underestimation of the unvaccinated population

One hypothesis might be an underestimation of the unvaccinated population in the ONS dataset. We have seen that the population included in this dataset does not cover the entire population of England recorded in the 2021 census. The population left out of the dataset is around 4,700,000 people. There would be a selection bias with systematic effects throughout the period if this proportion of the total population were not equally distributed between vaccinated and unvaccinated, and if it included a greater proportion of unvaccinated. We can see from the last two ONS reports that, although they cover a larger population than previous reports, the criteria for inclusion (and therefore exclusion) are the same as those based on the previous census. They are therefore subject to the same limitations. The main one is that the excluded population is not randomly selected, and therefore the population covered by the dataset is not representative of the general population. The key to inclusion is having a National Health Service (NHS) number. The probability of a vaccinated person not having an NHS number is virtually zero, because without a number you cannot be vaccinated. However, this is not the case for the unvaccinated, some of whom may not have been registered with a General Practitioner (GP) and therefore do not have an NHS number. As deaths among the unvaccinated are certified in the same way as those among the vaccinated, they cannot escape ONS registration. This would result in a relative overestimation of mortality rates for the unvaccinated and consequently an underestimation of RRs and SMRs for the vaccinated.

### 4.2 The healthy-vaccinee bias

Another possible contributory hypotheses is the so called healthy-vaccinee bias. ^16^^–^^19^ In 2021 a lower mortality among the vaccinated can also be explained partly by the healthy-adherer effect, or the healthy-vaccinee bias in the vaccination field. This effect is much more powerful than commonly thought: in fact, voluntary adherence to a treatment can be associated with a nearly halved mortality, ^20^^–^^22^ and even with a mortality reduction of 2.5 to 3 or more times ^16^^,^^18^^,^^23^^–^^25^ compared to the mortality of those who do not adhere. This effect is independent of the type of treatment to which one adheres voluntarily, being also found in randomized controlled trials in the placebo adherers (compared with placebo non-adherers). This effect may have several explanations. In the short time, individuals contingently ill tend to postpone vaccination. ^25^^,^^26^ In addition, doctors can renounce to vaccinate people considered close to death, whose subsequent death burdens the unvaccinated cohort disproportionately.

However, the healthy-adherer effect can be detected over many years, ^16^^,^^18^^,^^20^^,^^23^ because subjects adhering to preventive treatments are usually at the same time more likely to engage in healthy lifestyles than patients not adhering. ^17^^,^^21^ A healthy lifestyle includes diet, exercise, lower tobacco and alcohol consumption, less risky behaviors, ^27^ and the search for better health care. These features—difficult to capture in administrative databases—are associated with morbidity and mortality outcomes in observational studies. Moreover, the trust in the intervention to which one adheres can exert a beneficial placebo effect. The healthy-adherer bias is more difficult to correct than the opposite effect of confounding by indication (subjects in worse health conditions are vaccinated first), which is relatively easier to correct, provided it is known e.g. the number of comorbidities, or the Charlson comorbidity index of the groups to be compared. ^28^ Unfortunately, the UK ONS public data do not include any information about comorbidities. It is likely that the healthy-vaccinee bias effect will continue to operate to varying degrees in 2022 and 2023, albeit to a diminishing extent during periods in which vaccination mandates have been in force.

Moreover, it is plausible that the increase in the number of the vaccinated has diluted the opposite effect of confounding by indication. ^28^

A commonly used argument is the better known confounding by indication effect ^28^: it is likely that fragile subjects with multiple diseases have been vaccinated as a priority, followed by the others. However, as the vaccination campaigns proceed, the composition of the vaccinated and unvaccinated populations should result less unbalanced with respect to the pre-existing state of health. The ONS declare that “Changes in non-COVID-19 mortality by vaccination status are largely driven by the changing composition of the vaccination status groups. This is because of the priority given to clinically extremely vulnerable people or with underlying health conditions, and differences in timing of vaccination among eligible people". ^3^ However, a priority was also given to the healthier population of health workers. Moreover, the most fragile part of the population prioritized for the vaccination is a smaller portion (especially in the younger ages), and the composition of each age group progressively tends to be similar to that of the unvaccinated, in terms of general health conditions. Therefore, a decreasing trend would be expected, both because of the decreasing weight of the fragile fraction compared to the overall group and because of the harvesting effect, described below.

The healthy-vaccinee/un-healthy-un-vaccinee bias might be further supported by the web page by the Office for National Statistics, ^31^ showing that unvaccinated have higher tendency: to live in more deprived areas, urban areas, or social rented housing, to be not born in the UK or do not have English as a main language, to have never worked or to be long-term unemployed, to be more limited by a disability, and to be male (more men die than women). ^31^

### 4.3 The harvesting effect

The RRs of the first doses generally show high initial mortality peaks, possibly linked to the priority given to the fragile subjects. The early death of the most fragile causes that those who move on to the second dose are healthier overall. It is unfortunate that the last two ONS datasets do not provide data for the first three months of 2021, corresponding to the start of the vaccination programme. In fact, since vaccinations began with the older classes, in April 2021 the latter had not only completed the vaccination with the first dose, but had already started the second. Therefore our hypothesis can be confirmed only in the age groups 18-39 and 40-49, where one can clearly perceive the initial mortality peak of the first doses, probably already decreasing, followed by the initial mortality peak of the second doses, starting from lower values and with a lower maximum compared to that of the first doses.

As regards the third doses, the initial mortality peak disappears in all age groups, suggesting that many of the most “fragile” people have already died, and that a ‘healthy-vaccinee effect’ might partly explain the initial very low RR values.

Examining the SMR graphs, we note that, apart from the elderly, the points relating to all-cause deaths and non-COVID-19 deaths tend to overlap over time. This may indicate that the impact of COVID-19-related deaths vanishes, and that the risk of all-cause death and of deaths not related to COVID-19 is nearly the same. In fact, in the ONS dataset, approaching the end of the observation period, the COVID-19-deaths for the different vaccination statuses show an ever-increasing number of values indicated with <3, what prevents one from calculating both the RRs and the SMRs. This justifies the choice not to take into consideration COVID-19 deaths, but only all-cause and non-COVID-19 deaths.

Hence, the insistent push towards further vaccinations seems hardly motivated.

### 4.4 Loss of protective vaccine effectiveness and lower lethality of new variants

Again, examining the SMRs, the fact that initially the regression line of non-COVID-19 deaths is above that of all-cause deaths might indicate that the vaccine initially has a protective effect on COVID-19 deaths, thus lowering the risk of all-cause deaths, that include those COVID-19 related, among vaccinated people. The fact that they subsequently converge may indicate either that the vaccine gradually loses its protective effectiveness, or that the risk of COVID-19 deaths decreases due to the increasingly lower lethality of the new variants, or that the two causes act together.

The SMR graphs allow one to make a further consideration: in the 80-89 age group the convergence of the lines of all-cause deaths is much attenuated, and in the 90+ age group the lines are almost parallel, as one can see from the regression coefficients that differ less and less. This might indicate that COVID-19 still represents a risk for the elderly and that the vaccine therefore protects them by reducing the risk of all-cause deaths. Yet, it might also be due to the fact that the lower lethality of the new variants is offset by the fact that the vaccinated people get infected more than unvaccinated ^29^^–^^36^ and that by taking additional doses they are temporarily protected from the risk of dying from COVID-19.

### 4.5 Unintended effects of COVID-19 vaccines on the increasing deaths

Last but not least: why are the SMRs of non-COVID-19 related deaths increasing? Why should the risk of those vaccinated with any dose increase compared to that of the unvaccinated? Apart from the risk of immediate adverse reactions/events, the doubt naturally arises that the vaccine , might cause damage to the immune system, exposing the vaccinated to a greater risk of death from pathologies non-COVID-19 related ^37^^,^^38^

## 5. Limitation

Although this study shows an increase in SMR for all causes of death in vaccinated compared to unvaccinated individuals over time, we acknowledge some limitations. First, the lack of individual data published by the UK Office for National Statistics (ONS), which would have allowed us to determine the exact time at which each subject transitioned to a different vaccination status, did not allow us to use a different statistical approach that could provide more robust measures of association. Second, the extremely low baseline risk for all causes of death and death from non-COVID-19 is likely due to differences in the representativeness of comorbidity between groups, which the UK ONS does not provide (and likely does not possess to a large extent: see healthy-vaccinee bias). Therefore, it was not possible to adjust the estimation of the model for these covariates.

## 6. Conclusions

The English all-cause and non-COVID-19 mortality data by vaccination status, released by the UK ONS for the 26 months from April 2021 to May 2023, were analyzed by age group and vaccination status. Our findings show that all-cause deaths SMRs were increasing in any of the age groups considered. All-cause death SMRs, initially well below 1 for every age group, due to their increase, since a certain date exceeded the reference value of the unvaccinated people for the age groups 18-39, 80-89 and 90+. For the other age groups, it is possible to predict the date in which the SMR would reach the value 1, intersecting the unvaccinated level, provided that this trend is consistently maintained.

Non-COVID-19 SMR values show a very similar trend: initially they are much lower than 1, but it is not plausible such a vaccine protection from non-COVID-19 deaths. Therefore, this suggests significant biases in the ONS dataset, leading to an underestimation of the risks for the vaccinated. Regardless of the interpretative hypotheses, the fact that all-cause mortality SMRs in vaccinated increase over time compared to those of unvaccinated requires further, urgent investigation.

In any case, we hope that the ONS will resume the publication of the mortality data series by vaccination status, interrupted in May 2023, and that its example will be followed by other countries.

Moreover, the precautionary principle should suggest much greater caution in promoting extensive vaccination campaigns, pending the acquisition of valid explanations of the alarming phenomenon observed.

## Institutional review board statement

Not applicable.

## Informed consent statement

Not applicable.

## Author contributions

Conceptualization, M.A., G.M., G.D.P., M.C. and A.D.; methodology, formal analysis and writing—original draft preparation, M.A., G.M., G.D.P., M.C. and A.D.; writing—review and editing, M.A., G.M., G.D.P., M.C. and A.D. All authors have read and agreed to the published version of the manuscript.

## Disclaimer/publisher’s note

The statements, opinions and data contained in all publications are solely those of the individual author(s) and contributor(s) and not of MDPI and/or the editor(s). MDPI and/or the editor(s) disclaim responsibility for any injury to people or property resulting from any ideas, methods, instructions or products referred to in the content.

## Data Availability

The data presented in this study are openly available on the UK ONS web page entitled “Deaths by vaccination status, England”, available online: https://www.ons.gov.uk/peoplepopulationandcommunity/birthsdeathsandmarriages/deaths/datasets/deathsbyvaccinationstatusengland (accessed on 1 May 2024). Zenodo: **Supplementary Materials**, https://doi.org/10.5281/zenodo.13080361 ^39^ This project contains the following extended data:
•
Supplementary Tables 1-7 (population).xlsx
•
Supplementary Tables 15-21 (mortality non-Covid deaths).xlsx
•
Supplementary Tables 8-14 (mortality all causes).xlsx
•
Supplemetary figure.pptx
•
Table 2 Data are available under the terms of the Creative Commons Attribution 4.0 International license (CC-BY 4.0).
