# Supplementary material for: All-cause mortality according to COVID-19 vaccination status: An analysis of the UK office for National statistics public data
Source: F1000Res. 2025 Feb 20;13:886. Originally published 2024 Aug 5. [Version 2] doi: 10.12688/f1000research.154058.2 (PMC11868741; doi:10.12688/f1000research.154058.2)
Supplement: Peer review history

# Reviewer report: Reviewer response for version 2

**DOI:** 10.5256/f1000research.177063.r367845

**Published:** 2025-02-27

**Authors:** Jarle Aarstad

**Affiliations:** 1 HVL Business School, Western Norway University of Applied Science, Bergen, Norway

Dear authors,

 I now understand better the issue of standardized mortality ratio (SMR) among vaccinated against the corresponding unvaccinated groups and find the paper acceptable for indexing.

Is the work clearly and accurately presented and does it cite the current literature?

Partly

If applicable, is the statistical analysis and its interpretation appropriate?

Partly

Are all the source data underlying the results available to ensure full reproducibility?

Yes

Is the study design appropriate and is the work technically sound?

Partly

Are the conclusions drawn adequately supported by the results?

Yes

Are sufficient details of methods and analysis provided to allow replication by others?

Partly

Reviewer Expertise:

Applied statistical analysis.

I confirm that I have read this submission and believe that I have an appropriate level of expertise to confirm that it is of an acceptable scientific standard.

# Reviewer report: Reviewer response for version 1

**DOI:** 10.5256/f1000research.169038.r342218

**Published:** 2025-01-07

**Authors:** Stefan Pilz

**Affiliations:** 1 Medical University of Graz, Graz, Austria

This is an important retrospective study on the mortality rate according to vaccination status against SARS-CoV-2 in the UK. Based on publicly available national health data the authors show that mortality risk is, in general (for most time periods and age groups analyzed), higher in vaccinated versus unvaccinated individuals. SMRs in vaccinated individuals are lower than one in early periods of their observation period, but progressively increase over time and exceed one in several age groups suggesting that relative mortality risk progressively increases over time after vaccination. The discussion of these findings is well-written and includes several interesting views on potential biases in their findings. Overall, this is an interesting and important paper.

 I have only minor comments.

 The authors should aim to carefully explain very clearly the underlying analyses of their Tables and Figures so that they can also stand alone: e.g. in Table 1 it may not be clear for the reader that this is a regression analysis of SMRs and observation months (this has only been described in the text).

 The discussion is great, but a brief overall limitations section may improve the quality of the paper (e.g., retrospective study design, only age group data but no individual participant data, etc.). Several limitations have been nicely discussed but I would nevertheless suggest such a brief limitations section.

 The authors may check whether this article meets all the requirements of the STROBE guidelines. I do not request a STROBE guideline Table as a Supplement although this may be considered.

 The authors should carefully re-check the spelling and grammar of this article as there are some slight improvements required. E.g., Page 10, second paragraph, second line from the bottom “the risk of death” should be corrected to “the risk difference of death…”. In addition, some parts of the article may be difficult to read and understand for the reader. Consider improving this.

 In the Abstract the authors write that “the best way can be the analysis of all-cause mortality by vaccination”. I would rather be cautious to state “the best way” and re-phrase this to something like” Assessment of its real-world effect can be performed by analysis of all-cause mortality by vaccination status”.

 In the results section of the Abstract the last sentence is rather a discussion of the data than a results presentation. Consider revising and/or removing some parts to the discussion of the Abstract.

 Regarding data analyses and discussion it would be great (if possible depending on statistical power) to focus also on groups with vaccinations less than 21 days before, as for these groups hardly any (or only minimal) vaccine effectiveness on COVID-19 mortality can be assumed so that any mortality difference may well reflect some sort of bias. No problem if the authors are not willing to address this.

 Regarding the overall conclusions I would suggest being more cautious regarding strong statements (such as a moratorium on promoting mass vaccination campaigns should be implemented) as the findings are only observational and require further confirmation and more in-depth investigations at best with inclusion of more potential confounders and IPD.

Is the work clearly and accurately presented and does it cite the current literature?

Yes

If applicable, is the statistical analysis and its interpretation appropriate?

Yes

Are all the source data underlying the results available to ensure full reproducibility?

Yes

Is the study design appropriate and is the work technically sound?

Yes

Are the conclusions drawn adequately supported by the results?

Yes

Are sufficient details of methods and analysis provided to allow replication by others?

Yes

Reviewer Expertise:

Epidemiology and clinical research in endocrinology

I confirm that I have read this submission and believe that I have an appropriate level of expertise to confirm that it is of an acceptable scientific standard.

# Response

**Published:** 2025-01-14

**Authors:** Alberto Donzelli

**Affiliations:** Fondazione "Allineare Sanità e Salute", Italy

Dear Dr. Stefan Pilz,

 we would like to thank you for your suggestions. Although you have kindly indicated that the acceptance of your suggestions is at our discretion, we have decided willingly to include them in the text, because we believe they are useful for improving the article

 This is an important retrospective study on the mortality rate according to vaccination status against SARS-CoV-2 in the UK. Based on publicly available national health data the authors show that mortality risk is, in general (for most time periods and age groups analyzed), higher in vaccinated versus unvaccinated individuals. SMRs in vaccinated individuals are lower than one in early periods of their observation period, but progressively increase over time and exceed one in several age groups suggesting that relative mortality risk progressively increases over time after vaccination. The discussion of these findings is well-written and includes several interesting views on potential biases in their findings. Overall, this is an interesting and important paper.

 I have only minor comments.

**Q.** The authors should aim to carefully explain very clearly the underlying analyses of their Tables and Figures so that they can also stand alone: e.g. in Table 1 it may not be clear for the reader that this is a regression analysis of SMRs and observation months (this has only been described in the text).

**Author’s response**: Thank you for this advice. We have added a sentence that specifics this aspect.

**Q. **The discussion is great, but a brief overall limitations section may improve the quality of the paper (e.g., retrospective study design, only age group data but no individual participant data, etc.). Several limitations have been nicely discussed but I would nevertheless suggest such a brief limitations section.

**Author’s response:** Thank you for this advice. We have added the “Limitation” section.

**Q. **The authors may check whether this article meets all the requirements of the STROBE guidelines. I do not request a STROBE guideline Table as a Supplement although this may be considered.

**Author’s response**: Before submitting our paper, we checked the STROBE guidelines, but the most part of the items of the "STROBE Statements" are not applicable in our paper as we show a critical analysis of the data collected and provided by the ONS and the data analyzed was not collected by us. In the "Methods" section we described in detail what and how we used the information contained in the ONS dataset, but we cannot know how the ONS collected this data.

**Q** **. **The authors should carefully re-check the spelling and grammar of this article as there are some slight improvements required. E.g., Page 10, second paragraph, second line from the bottom “the risk of death” should be corrected to “the risk difference of death…”. In addition, some parts of the article may be difficult to read and understand for the reader. Consider improving this.

**Author’s response**: We inserted your suggestion.

**Q. **In the Abstract the authors write that “the best way can be the analysis of all-cause mortality by vaccination”. I would rather be cautious to state “the best way” and re-phrase this to something like” Assessment of its real-world effect can be performed by analysis of all-cause mortality by vaccination status”.

**Author’s response**: You are right, we have reworded the sentence according to your suggestion

**Q. **In the results section of the Abstract the last sentence is rather a discussion of the data than a results presentation. Consider revising and/or removing some parts to the discussion of the Abstract.

**Author’s response**: Thank you, we decided to revise and move this sentence in “Conclusion” section of the abstract.

**Q. **Regarding data analyses and discussion it would be great (if possible depending on statistical power) to focus also on groups with vaccinations less than 21 days before, as for these groups hardly any (or only minimal) vaccine effectiveness on COVID-19 mortality can be assumed so that any mortality difference may well reflect some sort of bias.

**Author’s response**: In the data analysis phase we also considered groups with vaccinations less than 21 days before, but it was not possible to perform any calculations as most of the boxes indicated <3 deaths.

 No problem if the authors are not willing to address this.

**Q. **Regarding the overall conclusions I would suggest being more cautious regarding strong statements (such as a moratorium on promoting mass vaccination campaigns should be implemented) as the findings are only observational and require further confirmation and more in-depth investigations at best with inclusion of more potential confounders and IPD.

**Author’s response**: Thank you, we did it

# Reviewer report: Reviewer response for version 1

**DOI:** 10.5256/f1000research.169038.r311454

**Published:** 2024-08-19

**Authors:** Jarle Aarstad

**Affiliations:** 1 HVL Business School, Western Norway University of Applied Science, Bergen, Norway

Dear Editor and authors,

 Thank you for giving me the opportunity to referee the manuscript entitled “All-cause mortality according to COVID-19 vaccination status: An analysis of the UK office for National statistics public data”.

 I find the article very interesting and illuminating, particularly comparing all-cause mortality with non-covid mortality, and along with a very good discussion, it increases the study’s validity and adds value to the study of covid-vaccines’ potential mortality effect.

 Concerning the Introduction, the authors relevantly address the research question and study objective. I believe, however, that the study by Mostert et al. (2024)(Ref-1) merits attention concerning excess mortality in Western countries.

 I assume the calculations are correct regarding the statistical analyses of the RRs CIs, but I recommend one or two references on which you base the calculations. Also, I did not understand what you mean by “standardized rate”. Altogether, I would like you to explain that part better, particularly for an audience unfamiliar with the approach, eventually in an appendix.

 I follow the logic in section 3.4, but I suspect you compare never vaccinated with ever vaccinated. Is that correct? I assume yes, but it was a bit unclear for me. In the Figures, you include the number of months, but would it be better to include their names? E.g., Apr. 2021, etc. In Figures 2, 3, 4, 5, and partly 6, the intersections are out of the sample, which you should state in the text.

 Very good and illuminating discussion. As you apply English/Welsh data, you may refer to the following web page by the Office for National Statistics, showing that unvaccinated tended to live in more deprived areas, urban areas, or social rented housing, were not born in the UK or did not have English as a main language, have never worked or are long-term unemployed, limited a lot by a disability, and are male (among you more men die than women). https://www.ons.gov.uk/peoplepopulationandcommunity/healthandsocialcare/conditionsanddiseases/articles/coronaviruscovid19latestinsights/vaccines

 Addressing the above mentioned issues adequately, I argue the study will meet the indexing standards.

 I wish you all the best when reviewing the manuscript.

 Sincerely,

 Referee 1.

Is the work clearly and accurately presented and does it cite the current literature?

Partly

If applicable, is the statistical analysis and its interpretation appropriate?

Partly

Are all the source data underlying the results available to ensure full reproducibility?

Yes

Is the study design appropriate and is the work technically sound?

Partly

Are the conclusions drawn adequately supported by the results?

Yes

Are sufficient details of methods and analysis provided to allow replication by others?

Partly

Reviewer Expertise:

Applied statistical analysis.

I confirm that I have read this submission and believe that I have an appropriate level of expertise to confirm that it is of an acceptable scientific standard, however I have significant reservations, as outlined above.

## References

[1] : Excess mortality across countries in the Western World since the COVID-19 pandemic: ‘Our World in Data’ estimates of January 2020 to December 2022. *BMJ Public Health* .2024;2(1) : 10.1136/bmjph-2023-000282 10.1136/bmjph-2023-000282

# Response

**Published:** 2024-08-29

**Authors:** Alberto Donzelli

**Affiliations:** Fondazione "Allineare Sanità e Salute", Italy

Authors’ responses

 Dear Dr. Jarle Aarstad,

 We want to thank you for your advice which contribute to improve our paper. Below the answers to your questions.

**Reviewer response: **Dear Editor and authors,

 Thank you for giving me the opportunity to referee the manuscript entitled “All-cause mortality according to COVID-19 vaccination status: An analysis of the UK office for National statistics public data”.

 I find the article very interesting and illuminating, particularly comparing all-cause mortality with non-covid mortality, and along with a very good discussion, it increases the study’s validity and adds value to the study of covid-vaccines’ potential mortality effect.

 Concerning the Introduction, the authors relevantly address the research question and study objective. I believe, however, that the study by Mostert et al. (2024)(Ref-1) merits attention concerning excess mortality in Western countries.

**Author’s response**: Thank you for your valuable feedback. We have added the reference in the "Introduction" section.

**Reviewer response: **I assume the calculations are correct regarding the statistical analyses of the RRs CIs, but I recommend one or two references on which you base the calculations. **Author’s response**: Sorry, but we think do not understand, the reference regarding the statistical analyses of the RRs Cis are indicated just before the colons in the sentence above the formula and they are mentioned as 7 and 8 references. 

**Reviewer response: **Also, I did not understand what you mean by “standardized rate”. **Author’s response**: Sorry, you are right. For “standardized rate” we mean the Age-standardized mortality rates per 100,000 person-years, standardized to the 2013 European Standard Population using five-year age groups from those aged 10 years and over as defined in "Note 1" of the spreadsheet called "Notes" of the ONS dataset. We changed the sentence.

**Reviewer response: **Altogether, I would like you to explain that part better, particularly for an audience unfamiliar with the approach, eventually in an appendix.

**Author’s response**: Sorry but we are not sure we understand, could you possibly be more specific? We inserted all the steps of the calculations performed by us, and we are not sure what other steps we could describe in a further appendix. However, we also interpreted your perplexity as due to the fact that we have inserted a bibliographic reference in Italian, less useful to international researchers. We have therefore replaced the current entry n. 9 with the same reference text in English.

**Reviewer response: **I follow the logic in section 3.4, but I suspect you compare never vaccinated with ever vaccinated. Is that correct? I assume yes, but it was a bit unclear for me. **Author’s response**: Exactly, you are correct, we used the same terminology used in in the Table 2 of the ONS dataset.

**Reviewer response: **In the Figures, you include the number of months, but would it be better to include their names? E.g., Apr. 2021, etc.

**Author’s response**: Thank you, we replaced the numbers with the names.

**Reviewer response: **In Figures 2, 3, 4, 5, and partly 6, the intersections are out of the sample, which you should state in the text.

**Author’s response**: Sorry, you are right. We added a sentence that specifics this aspect.

**Reviewer response: **Very good and illuminating discussion. As you apply English/Welsh data, you may refer to the following web page by the Office for National Statistics, showing that unvaccinated tended to live in more deprived areas, urban areas, or social rented housing, were not born in the UK or did not have English as a main language, have never worked or are long-term unemployed, limited a lot by a disability, and are male (among you more men die than women). https://www.ons.gov.uk/peoplepopulationandcommunity/healthandsocialcare/conditionsanddiseases/articles/coronaviruscovid19latestinsights/vaccines

 Addressing the above mentioned issues adequately, I argue the study will meet the indexing standards. I wish you all the best when reviewing the manuscript.

**Author’s response**: Thank you for this advice. We have added a reflection on this aspect in the “Discussion” section.

# Response

**Published:** 2025-01-14

**Authors:** Alberto Donzelli

**Affiliations:** Fondazione "Allineare Sanità e Salute", Italy

Dear Dr. Jarle Aarstad,

 We want to thank you for your advice which contribute to improve our paper. Below are the answers to your questions.

 Dear Editor and authors,

**Thank you for giving me the opportunity to referee the manuscript entitled “All-cause mortality according to COVID-19 vaccination status: An analysis of the UK office for National statistics public data”.**

**I find the article very interesting and illuminating, particularly comparing all-cause mortality with non-covid mortality, and along with a very good discussion, it increases the study’s validity and adds value to the study of covid-vaccines’ potential mortality effect.**

**Q.** Concerning the Introduction, the authors relevantly address the research question and study objective. I believe, however, that the study by Mostert et al. (2024)(Ref-1) merits attention concerning excess mortality in Western countries.

**Author’s response**: Thank you for your valuable feedback. We have added the reference in the "Introduction" section.

**Q.** I assume the calculations are correct regarding the statistical analyses of the RRs CIs, but I recommend one or two references on which you base the calculations.

**Author’s response**: sorry, but we think do not understand, the reference regarding the statistical analyses of the RRs CIs are indicated just before the colons in the sentence above the formula and they are mentioned as 8 and 9 references. 

**Q** **.** Also, I did not understand what you mean by “standardized rate”.

**Author’s response**: sorry, you are right. For “standardized rate” we mean the Age-standardised mortality rates per 100,000 person-years, standardised to the 2013 European Standard Population using five-year age groups from those aged 10 years and over as defined in "Note 1" of the spreadsheet called "Notes" of the ONS dataset. We changed the sentence.

**Q** **.** Altogether, I would like you to explain that part better, particularly for an audience unfamiliar with the approach, eventually in an appendix.

**Author’s response**: sorry but we think do not understand, could you be more specific? We inserted all the steps of the calculations performed by us and we would not know which other steps we could describe in a further appendix.

**Q** **.** I follow the logic in section 3.4, but I suspect you compare never vaccinated with ever vaccinated. Is that correct? I assume yes, but it was a bit unclear for me.

**Author’s response**: exactly, you are correct, we used the same terminology used in the Table 2 of the ONS dataset.

**Q.**In the Figures, you include the number of months, but would it be better to include their names? E.g., Apr. 2021, etc. **Author’s response**: thank you, we replaced the numbers with the names.

**Q** **.** In Figures 2, 3, 4, 5, and partly 6, the intersections are out of the sample, which you should state in the text.

**Author’s response**: sorry, you are right. We added a sentence that specifics this aspect.

**Q. **Very good and illuminating discussion. As you apply English/Welsh data, you may refer to the following web page by the Office for National Statistics, showing that unvaccinated tended to live in more deprived areas, urban areas, or social rented housing, were not born in the UK or did not have English as a main language, have never worked or are long-term unemployed, limited a lot by a disability, and are male (among you more men die than women). https://www.ons.gov.uk/peoplepopulationandcommunity/healthandsocialcare/conditionsanddiseases/articles/coronaviruscovid19latestinsights/vaccines

 Addressing the above mentioned issues adequately, I argue the study will meet the indexing standards.

**Author’s response**: thank you for this advice. We have added a reflection on this aspect in the “Discussion” section.

 I wish you all the best when reviewing the manuscript.
